# Impact of a prevention educational program of central line-associated bloodstream infection in a polivalent ICU

**DOI:** 10.1186/2197-425X-3-S1-A1015

**Published:** 2015-10-01

**Authors:** L Navarro Guillamon, MM Jimenez Quintana, F Manzano Manzano

**Affiliations:** Granada, Complejo Hospitalario, Granada, Spain

## Introduction

The decline in the central line-associated bloodstream infection (CLABSI) rate has become a priority for intensive care units.

## Objective

To determine whether an educational intervention program can reduce the incidence of CLABSI in patients admitted to a polivalent ICU.

## Methods

A retrospective, interventional, quasi-experimental study conducted in a medical-surgical ICU in two periods (4/1/2005 - 30/ 6/2009 and 4/1/2010 - 31/12/2012), including all patients admitted to our ICU. The intervention since September 2009 includes: proper hands hygiene, use of chlorhexidine, total aseptic measures during the central venous canalization, preferential use of subclavian vein and removal of useless central venous catheter (CVC). The main outcome variable was the rate of CLABSI (infections / 1000 days of catheter) before and after the intervention. Data were collected according ENVIN-HELICS study. Other variables included demographics data, primary diagnosis, APACHE II score, need of mechanical ventilation, use and duration of CVC, hospital mortality, and others.

Statistical analysis: Descriptive, chi-square, t-Student, multivariate logistic regression analysis.

## Results

622 and 108 patients respectively were studied, without significant differences in demographic variables between the two groups, except for the APACHE II score (16.89 ± 7.7 and 19.3 ± 9.2, p < 0.001). The incidence density rate of CLABSI in the interventional period compared to the pre-intervention period was 2.18 vs 3.98 / 1000 days of CVC (p = 0.045). The CVC were used in 83.9% vs 98.2% (p = 0.01); and the duration was 10 ± 16 days vs 8.73 ± 10 days (p = 0.09), respectively. The factors statistically associated with the onset of CLABSI in the multivariate analysis were period of intervention (OR 0.12, 95% CI 0.09 to 0.91, p = 0.041) and the duration of CVC placement (OR 1.08, 95% CI 1.04-1.11, p < 0.001 ).

## Conclusion

These interventions have demonstrated a positive impact in reducing the number of CLABSI, despite the increase in the duration of CVC placement in the intervention period.

